# Determination of microbiological characteristics in the digestive tract of different ruminant species

**DOI:** 10.1002/mbo3.769

**Published:** 2018-12-25

**Authors:** Tian Zhang, Yingyu Mu, Deqing Zhang, Xueyan Lin, Zhonghua Wang, Qiuling Hou, Yun Wang, Zhiyong Hu

**Affiliations:** ^1^ College of Animal Science Shandong Agricultural University Taian China; ^2^ Laboratory Department Taian Central Hospital Taian China

**Keywords:** bacterial community, bacterial diversity, rumen fluid, rumen species

## Abstract

Holstein dairy cows, Chinese Luxi Yellow cattle, Chinese Laoshan dairy goats, and Chinese Bohai Black cattle were selected for the study. The 16S rDNA sequencing technique was used to analyze the microflora in the digestive tract. The rumen flora in high milk‐yield Holstein dairy cows showed significantly higher proportions of *Treponema, Butyrivibrio*,* Coprococcus*,* Shuttleworthia*,* Lachnospira,* and *Selenomon*as, compared with the rumen flora in Chinese Bohai Black cattle and Chinese Luxi Yellow cattle (*p* < 0.05). In addition, the abundances of *Succiniclasticum, Ruminococcus,* and *Fibrobacter* in the rumen fluid of high‐yield dairy cows were significantly higher than those in rumen flora of dairy goats. Compared with ruminal flora in Chinese Luxi Yellow cattle, the rumen flora in high‐yield dairy cattle showed significantly higher *Prevotella*. Compared with the rumen flora in Chinese Laoshan dairy goats, Chinese Bohai Black cattle, and Chinese Luxi Yellow cattle, the flora in high‐yielding dairy cows showed significantly lower proportions of CF231, 02d06, *Oscillospira*, RFN20, *Desulfovibrio*,* Methanobrevibacter,* and SHD‐231. In addition, compared with the rumen flora in dairy goats, the rumen flora in high‐yielding dairy cattle displayed significantly lower proportion of *Enterococcus*. Compared with the rumen flora in Chinese Bohai Black cattle, the flora in high‐yielding dairy cattle exhibited significantly lower *Ruminococcus*, YRC22, *Pseudobutyrivibrio*, L7A_E11, BF311, p‐75‐a5, and *Dehalobacterium*. Compared with the rumen flora in Chinese Luxi Yellow cattle, the flora in the high‐yield dairy cows also displayed significantly lower proportions of *Ruminococcus*, YRC22, BF311, *Paludibacter,* and *Dehalobacterium*.

## 1 INTRODUCTION

Improving the feed conversion rate is critical for improving the international competitiveness of the Chinese dairy industry; in particular, due to the shortage of high‐quality roughage, it is important to improve the conversion rate of low‐quality roughage to improve dairy cattle yield and reduce production costs. Previous studies have shown that microorganisms are very important for improving efficiency of roughage feed conversion (Wang, 2011). It is envisaged that the signature microorganisms in local ruminants that can tolerate roughage can be used to improve the roughage feed conversion efficiency of dairy cows which require fine‐tuned feeding regimens for production and reduce the cost per unit yield. The local ruminants that tolerate roughage in Shandong Province include Chinese Laoshan dairy goats, Chinese Bohai Black cattle, and Chinese Luxi Yellow cattle.

Chinese Laoshan dairy goats originated from Jiaodong Peninsula of Shandong Province and are mainly distributed in the Laoshan and surrounding areas. They are a high‐quality, high‐yield local breed created after years of breeding in Laoshan area and are one of the China's finest breeds of dairy goats. Chinese Laoshan dairy goats are characterized by adaptability, resistance to coarse feeding, rapid growth and development, strong stature, high milk production, and stable genetic performance; they are inexpensive and are suited to grazing in mountainous, hilly, and plains terrain. The breed was listed in “Records of China Livestock Breeds” in 1998. Chinese Laoshan dairy goats’ milk production lasts for a period of 8–10 months, with an average milk yield of 340 kg for the first birth, 600 kg for the second birth, and 700 kg for the third birth (Sheng, 2014). The highest annual milk production can be as high as 1,300 kg. The fresh milk has a density of 1.028 g/ml and contains 12.03% dry matter, 2.89% milk protein, 3.73% milk fat, and 4.53% lactose, as well as high levels of methionine, lysine, and histidine. Previous research on Laoshan dairy goats was mainly focused on dietary energy, protein levels, and the effects of certain genes on lactation and production traits (Song, Wang, Cheng, Dai, & Lin, 2015; Wang et al., 2012; Yu et al., 2015). There are few researches on the role of ruminal microorganisms under coarse feeding conditions.

Chinese Bohai Black cattle belong to a rare variety of black‐coated cattle of Bovidae. This is one of the three major black‐haired cattle varieties in the world. The Cattle Breeding Committee of China designated Bohai Black as one of the China's eight famous cattle breeds. Traditionally, this breed has been called Bohai Black because its whole body is black. This fine breed resulted from long‐term domestication and selection in Huan Bohai County of Shandong Province. Bohai Black is a unique, medium‐sized local breed used for meat and service and was created by long‐term selective breeding. It is resistant to coarse feed and disease, and it has good adaptability and genetic stability (Liu, 2015).

The Luxi cattle breed is a fine variety of meat livestock unique to Shandong Province. They have a strong ability to digest hay, straw, and other rough feed. Cellulose is composed of 36 single chains forming a highly crystalline microfibril structure with intrachain and interchain hydrogen bonds, van der Waals force, and hydrophobic force. The rumen microbial system of Luxi cattle can efficiently degrade plant cellulose substrates. It is speculated that this efficient mechanism may primarily depend on the various glycoside hydrolases contained in the rumen of the cattle, which are produced by a variety of symbiotic microorganisms in the rumen. *Ruminococcus* produces fibrous bodies with a large molecular weight and a variety of glycoside hydrolase activities. In addition, we speculate that *Ruminococcus* may gradually degrade the natural plant cellulose substrate by rolling a large number of fibrous bodies on the surface to destroy the highly crystalline cellulose chains (Wang, 2011). *Ruminococcus*–*fibrous* body complexes may be necessary to degrade crystalline cellulose. Fibrous bodies that are free from *Ruminococcus* cells will lose most of their ability to degrade natural plant cellulose (Wang, 2011).

Chinese Laoshan dairy goats, Bohai Black, and Luxi cattle can undergo long‐term adaptation to rough forage and a rough environment. Their digestive tract may have unique or dominant microorganisms that improve the utilization of poor‐quality roughage. Therefore, this study is aimed at determining differences and similarities of ruminal microbial composition among high‐performance Holstein dairy cows, Chinese Laoshan dairy goats, Chinese Bohai Black cattle, and Luxi cattle; it attempts to determine the typical characteristics of various types of ruminant intestinal flora. It is hoped that these microorganisms in ruminants that can tolerate poor roughage can be used in the dairy cattle industry to improve the effectiveness of dairy cows using local poor‐quality feed resources and to improve lactation performance for unit cost reduction. This study will provide a vital reference for screening, purification, and identification of bacteria that impart tolerance to roughage and to further researches into microbial nutrition control.

## 2 MATERIALS AND METHODS

### 2.1 Animals

The study included six healthy high‐yield (milk production more than 30 kg) and six low‐yield (milk production <20 kg) Holstein dairy cows, five Chinese Laoshan milk goats, six Chinese Luxi beef cattle, and six Chinese Bohai Black beef cattle. Holstein dairy cows were paired for similar age, parity, and nearing lactation days. Chinese Laoshan milk goats, Chinese Luxi beef cattle, and Chinese Bohai Black beef cattle were healthy.

### 2.2 Sample collection

Representative rumen fluid samples were obtained from all experimental animals via the animals’ mouth with the oro‐ruminal sampling device within two hours prior to morning feeding. The collected rumen fluid was dispensed into three 50‐ml sterile centrifuge tubes. All samples were immediately flash‐frozen in liquid nitrogen and stored at −80°C for future testing.

### 2.3 Experimental procedures for 16S rDNA sequencing

The samples were slowly thawed at 4°C. Total DNA was extracted from the rumen fluid samples using the Stool DNA Isolation kit (Tiangen, Beijing, China). DNA samples were quantified using a NanoDrop spectrophotometer (Nyxor Biotech, Paris, France) and then transferred to BGI Genomics for V4 region of the 16S rDNA gene sequencing with PE250 Miseq. The PCR primer used for 16S rDNA amplicon libraries was 515F‐806R.

### 2.4 Bioinformatics analysis for 16S rDNA sequencing

The raw data were filtered to eliminate the adapter pollution and low‐quality reads to obtain clean sequences (Douglas et al., 2014). Sequence reads with an average quality of under 20 over a 30‐bp sliding window as per the phred algorithm were truncated, and trimmed reads having less than 75% of their original length, as well as its paired read, were removed. Then, paired‐end reads with overlap were merged into tags using FLASH (Magoc & Salzberg, 2011; Fast Length Adjustment of Short reads, v1.2.11). Tags were clustered to OTU at 97% sequence similarity by scripts of software USEARCH (v7.0.1090; Edgar, 2013). Chimeras were filtered out by using UCHIME (v4.2.40; Edgar, Haas, Clemente, Quince, & Knight, 2011). OTU representative sequences were taxonomically classified using the Ribosomal Database Project (RDP) Classifier v.2.2 (Cole et al., 2014) trained on the Greengenes database (V201305; DeSantis et al., 2006) using 0.5 confidence values as cutoff thresholds. Below this, confidence level is classified as unclassified bacteria and OTUs were filtered to remove unassigned OTUs.

VennDiagram and package “ade4” of software R (v3.0.3) were used separately in Venn diagram and OTU PCA analysis. The tag numbers of each taxonomic rank (phylum, class, order, family, genus, and species) or OTU in different samples were summarized in a profiling table. The species with abundances of less than 0.5% were classified into “others” in other ranks for all samples.

The representative sequences were aligned against the Silva core set (Silva_108_core_aligned_seqs) using PyNAST by “align_seqs.py.” The indices of alpha diversity were calculated by Mothur (v1.31.2), and the corresponding rarefaction curves were drawn by R (v3.0.3). The Wilcoxon rank‐sum test was used for comparison of two groups using the alpha‐diversity indices. Beta‐diversity analysis was done by software QIIME (v1.80; Caporaso et al., 2010). Principal coordinate analysis (PCoA) was used to exhibit the differences between the samples according to the matrix of beta‐diversity distance.

Metastats (https://metastats.cbcb.umd.edu/) via a *T* test on the species abundance data between groups obtains *p*‐values and corrects the *p*‐values to obtain *q*‐values. Finally, based on the *p*‐value (or *q*‐value), the species that cause differences in the composition of the two groups were screened, *p* ≥ 0.05.

## 3 RESULTS

### 3.1 Sequence stitching

Paired‐end reads were stitched into contigs based on their overlap. A total of 2,733,352 contigs were obtained, an average of 94,253 contigs per sample. The *SD* value was 30,677. The contigs had an average length of 252 bp and an *SD* value of 0 bp.

### 3.2 OTU AND abundance

#### 3.2.1 OTU statistics

After the stitched Tags are optimized, they were clustered at 97% similarity for operational taxonomic units (OTUs) to classify species. The abundance information of each sample in each OTU was counted, and the abundance of OTU preliminarily indicated the species richness of the sample. A total of 4,314 OTUs were found in 29 samples.

#### 3.2.2 OTU Venn chart analysis

**Figure 1:**
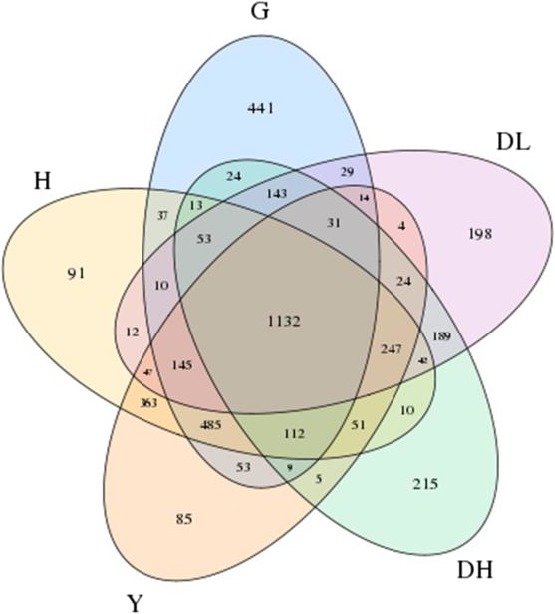
Shared OTU across different samples or groups

At a similarity level of 97%, the OTU number in each sample was obtained. The Venn chart (Figure 1) depicts the number of OTUs unique to each sample or shared among multisamples and intuitively displayed OTU overlap among samples. There were 2,300 OTUs in total found in the rumen fluid of the high milk‐yield Holstein dairy cows, 2,320 OTUs in the rumen fluid of the low‐yield Holstein dairy cows, 2,731 OTUs identified from the rumen fluid of dairy goat, 2,850 OTUs from Bohai Black, and 2,807 OTUs from Luxi Yellow.

#### 3.2.3 PCA of OTUs

**Figure 2:**
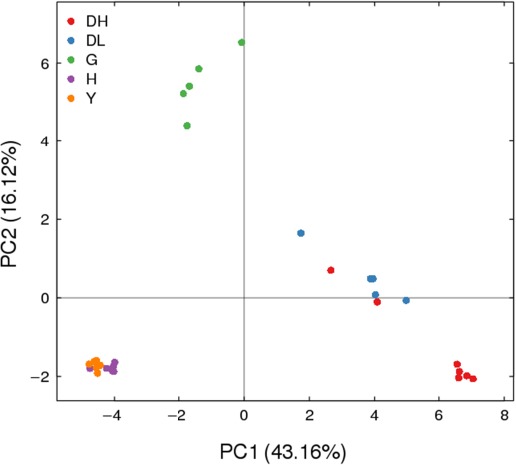
PCA based on OTU abundance

As shown in Figure 2, the Chinese Bohai Black cattle group was very close to the Chinese Luxi Yellow cattle group in terms of PCA results in the graph. These two groups contained similar OTUs, whereas the other two groups of samples distantly scattered; this indicated that the OTU compositions among the other groups were quite different.

### 3.3 Taxonomic annotation

**Figure 3:**
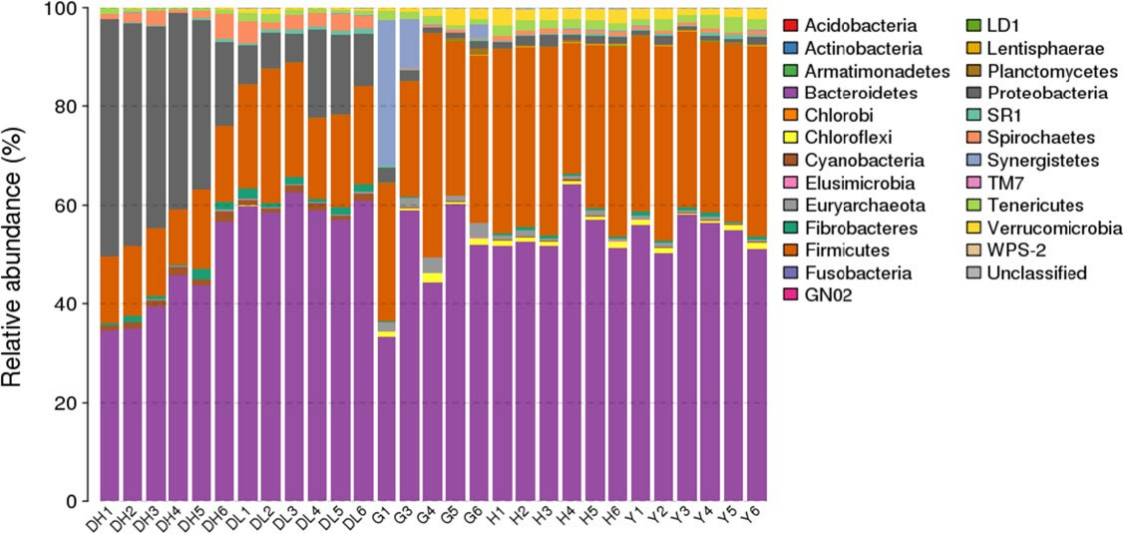
Phylum‐level taxonomic composition distribution in samples

It can be seen from Figure 3, that at the phylum level, the high‐yield dairy cow rumen samples contained 21 phyla of bacteria. The phyla that contributed more than 1% to the microflora included *Bacteroidetes*,* Proteobacteria*,* Firmicutes*,* Spirochaetes*,* Cyanobacteria*, and *Fibrobacteres*. The low‐yield dairy cow samples contained 22 phyla, among which the phyla that contributed 1% or more included *Bacteroidetes*,* Firmicutes*,* Proteobacteria*,* Spirochaetes*,* Cyanobacteria*, and *Fibrobacteres*. The dairy goat rumen samples contained 24 phyla; among these, the phyla that contributed more than 1% included *Bacteroidetes*,* Firmicutes*,* Synergistetes*,* Euryarchaeota*,* Proteobacteria*,* Verrucomicrobia*,* Tenericutes,* and *Chloroflexi*. The Bohai Black cattle rumen samples contained 24 phyla, and among these, the phyla that contributed 1% or more included *Bacteroidetes*,* Firmicutes*,* Verrucomicrobia*,* Tenericutes,* and *Proteobacteria*. The Luxi cattle rumen samples contained 25 phyla, and among these, the phyla that contributed more than 1% included *Bacteroidetes*,* Firmicutes*,* Tenericutes*,* Verrucomicrobia,* and* Proteobacteria*.

**Figure 4:**
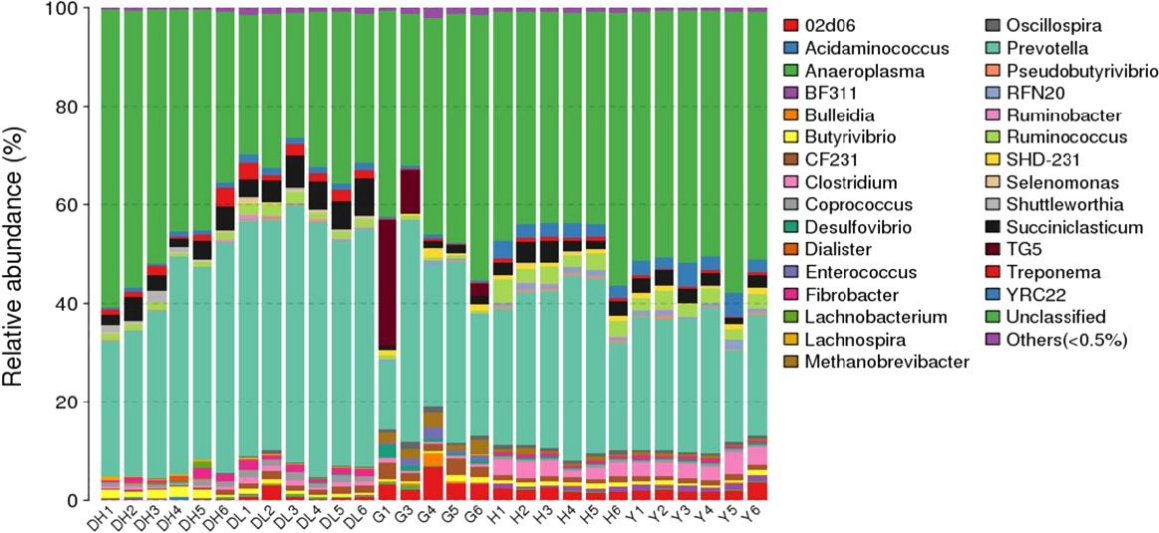
Genus‐level taxonomic composition distribution in samples

As shown in Figure 4, at the genus level, 92 genera were detected in high‐yield cow rumen samples. Among them, the genera contributed more than 0.5% included *Prevotella, Succiniclasticum, Treponema, Butyrivibrio, Ruminococcus, Fibrobacter,* YRC22*, Coprococcus,* and* Shuttleworthia*. A total of 88 genera were found in the rumen fluid of low‐yield cows, among which, the genera that contributed more than 0.5% included *Prevotella, Succiniclasticum, Treponema, Ruminococcus,* YRC22*, Sclerotella,* CF231*, Fibrobacter,* 02d06, and* Butyrivibrio*.

A total of 113 genera were found in rumen fluid of dairy goats; among these, the genera that contributed than 0.5% included *Prevotella,* TG5, 02d06, CF231,* Methanobrevibacter, Enterococcus, Succiniclasticum,* SHD‐231,* Desulfovibrio, Oscillospira, Butyrivibrio, Bulleidia, Ruminococcus,* and YRC22 (Figure 4).

A total of 110 genera were detected in the rumen fluid of Chinese Bohai Black cattle; of these, the genera that contributed 0.5% or more included *Prevotella, Ruminococcus, Succiniclasticum,* YRC22, 02d06, RFN20, CF231*,* SHD‐231,* Butyrivibrio, Treponema,* BF311,* Oscillospira, Methanobrevibacter, Ruminococcus,* and* Fibrobacter* (Figure 4).

A total of 98 genera were found in the rumen fluid of Luxi cattle; of these, the genera that contributed 0.5% or more included *Prevotella,* YRC22*, Ruminococcus, Succiniclasticum,* 02d06, CF231, BF311, RFN20, SHD‐231*, Fibrobacter, Butyrivibrio, Oscillospira, Coprococcus,* and* Treponema* (Figure 4).

### 3.4 Sample diversity analysis

#### 3.4.1 Single sample diversity analysis

**Figure 5:**
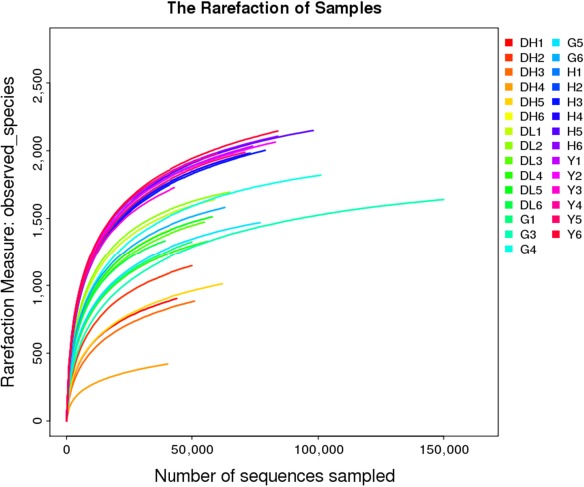
Sample‐based rarefaction curve of observed species indices

Figure 5 shows the corresponding rarefaction curves of the observed species index. It was found that the rarefaction curves of all samples eventually plateaued, indicating that the sequencing depth was sufficient and there was substantial coverage of all the species in the sample.

**Figure 6:**
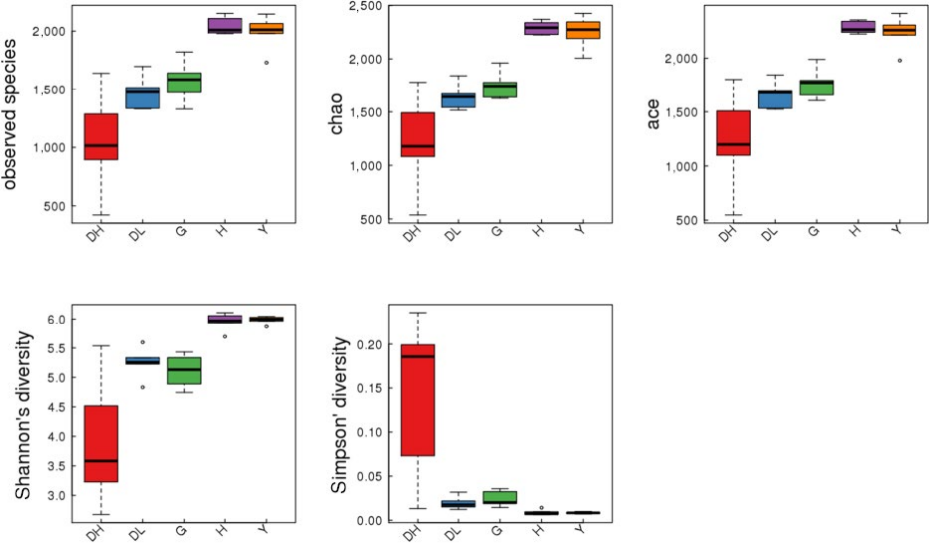
Boxplot of alpha‐diversity indices among groups

The observed species index, the chao index, and the ACE index reflect the richness of the community in the sample. The Shannon index and the simpson index reflect the diversity of the community and are affected by species richness and species uniformity in the sample community. Figure 6 shows boxplots of microflora in the rumen fluid of high‐yielding dairy cows, low‐yielding dairy cows, dairy goats, Bohai Black cattle, and Luxi cattle. There were significant differences in species richness and evenness. The diversity of rumen bacteria in high‐yield dairy cows was significantly lower than that in low‐yielding dairy cows, dairy goats, Bohai Black cattle, and Luxi cattle.

#### 3.4.2 Comparison of diversity between samples

**Figure 7:**
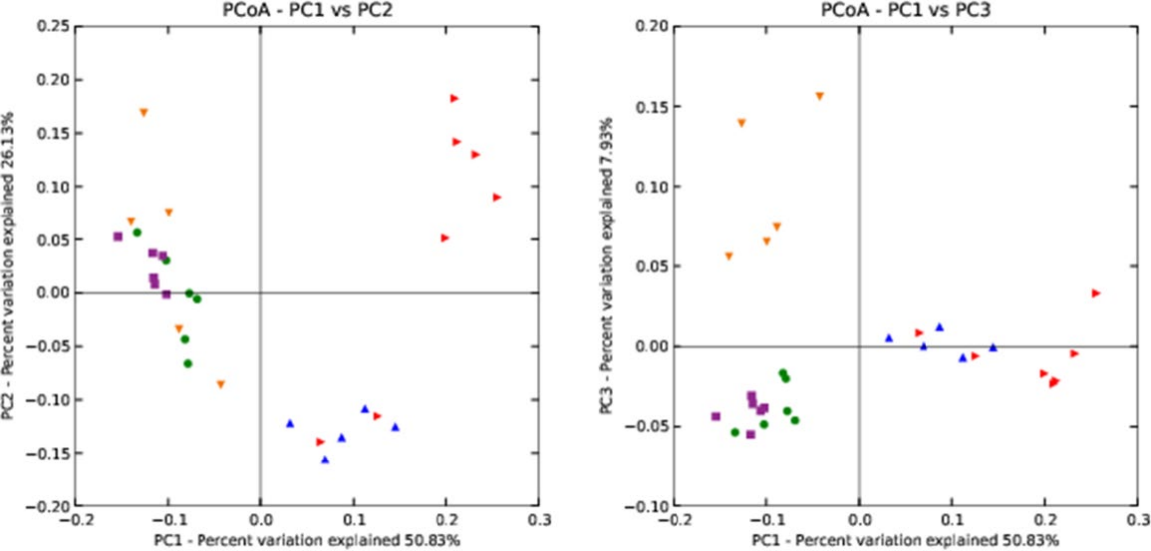
Weighted UniFrac PCoA results for all samples. High‐yield dairy cows, low‐yield dairy cows, milk goats, Bohai Black, and Luxi cattle were, respectively, represented by red triangles, blue triangles, orange triangles, green dots, and purple squares

The results of PCoA are shown in Figure 7. The PC1 axis explained 50.83% of the variability, the PC2 axis explained 26.13% variance, and the PC3 axis explained 7.93% variability. These results clearly showed distinctions among the microbial compositions in the rumen fluid of high‐yield dairy cows, low‐yield dairy cows, dairy goats, Bohai Black cattle, and Luxi cattle.

#### 3.4.3 Significant differences between sample groups

##### Phylum level

A total of 0.06% bacteria in the rumen fluid of high‐yield cows remained unidentified at the phylum level. The percentage of unidentified bacteria was 0.11% for samples from the low‐yield dairy cows, 0.13% for samples from Chinese Laoshan dairy goats, 0.28% for samples from Chinese Bohai Black cattle, and 0.27% for samples from Luxi cattle (Table 1).

**Table 1:** Significant differences in the flora of rumen fluids of high‐production dairy cows and low production dairy cows at the phylum level

Phylum	Mean (HighS)	*SE* (HighS)	Mean (LowS)	*SE* (LowS)	*p*‐Value	FDR
Bacteroidetes	43.72	3.01	58.00	1.25	0.003	0.010
Proteobacteria	33.21	4.73	11.40	1.27	0.001	0.010
SR1	0.26	0.09	0.76	0.11	0.002	0.010
Verrucomicrobia	0.18	0.07	0.60	0.14	0.012	0.031
Euryarchaeota	0.10	0.02	0.22	0.03	0.008	0.024
Planctomycetes	0.04	0.01	0.11	0.01	0.003	0.010
Synergistetes	0.02	0.01	0.05	0.00	0.002	0.010
Chloroflexi	0.01	0.00	0.04	0.01	0.002	0.010

Note

*p* < 0.05 indicates significant difference. HighS denotes high‐yielding dairy cow rumen fluid. LowS denotes low‐yielding dairy cow rumen fluid.

Compared with samples of low‐yield dairy cows, the samples of high‐yield cows contained a significantly greater proportion of *Proteobacteria* (*p* < 0.05) and a significantly lower proportions of *Bacteroidetes*, SR1, *Verrucomicrobia*,* Euryarchaeota*,* Planctomycetes*,* Synergistetes,* and *Chloroflexi* (*p* < 0.05; Table 1).

It can be seen from Table 2 that the relative contents of *Proteobacteria*,* Spirochaetes*,* Cyanobacteria,* and *Fibrobacteres* in the rumen of high‐yield dairy cows were significantly higher than those in the rumen fluid of Chinese Laoshan dairy goats (*p* < 0.05). The relative contents of *Firmicutes*,* Tenericutes*,* Verrucomicrobia*,* Euryarchaeota*,* Chloroflexi*,* Actinobacteria*, and *Lentisphaerae* were significantly lower in the rumen fluid of Chinese Laoshan dairy goats (*p* < 0.05). *Armatimonadetes* was found only in the rumen fluid Chinese Laoshan dairy goats and not found in the rumen fluid of high‐yield dairy cattle.

**Table 2:** Significant differences in rumen fluid flora between high‐production dairy cows and goats at the phylum level

Phylum	Mean (DH)	*SE* (DH)	Mean (G)	*SE* (G)	*p*‐Value	FDR
Proteobacteria	33.2399	5.7075	1.6983	0.3845	0.0003	0.0023
Firmicutes	15.0857	1.1441	32.3503	3.6730	0.0011	0.0054
Spirochaetes	2.5066	0.6484	0.2323	0.0471	0.0039	0.0116
Cyanobacteria	1.4165	0.1463	0.0960	0.0213	0.0001	0.0020
Fibrobacteres	1.1374	0.2936	0.0329	0.0049	0.0027	0.0091
Tenericutes	0.8319	0.1803	1.3730	0.1597	0.0413	0.0902
Verrucomicrobia	0.2051	0.0898	1.6812	0.4987	0.0139	0.0371
Euryarchaeota	0.1030	0.0301	2.2288	0.3762	0.0002	0.0023
Chloroflexi	0.0125	0.0090	1.0101	0.2502	0.0018	0.0073
Actinobacteria	0.0073	0.0019	0.0903	0.0169	0.0006	0.0035
Lentisphaerae	0.0042	0.0023	0.0300	0.0103	0.0308	0.0740
Armatimonadetes	0.0000	0.0000	0.0078	0.0036	0.0471	0.0943

Note

*p* < 0.05 indicates a significant difference. DH denotes high‐yield dairy cow rumen fluid, while G denotes dairy goat rumen fluid.

It can be seen from Table 3 that the rumen fluid in high‐yield dairy cows contained significantly higher levels of* Proteobacteria*,* Spirochaetes,* and *Cyanobacteria* than rumen fluid of Chinese Bohai Black cattle (*p* < 0.05). The relative contents of *Bacteroidetes*,* Firmicutes*,* Tenericutes*,* Verrucomicrobia*,* Euryarchaeota*,* Planctomycetes*,* Synergistetes*,* Chloroflexi*,* Actinobacteria*, WPS‐2,* Lentisphaerae,* and LD1 were significantly lower in the rumen fluid of the high‐yield dairy cows than in the rumen fluid of Chinese Bohai Black cattle (*p* < 0.05). The rumen fluid of Chinese Bohai Black cattle contained low abundance *Armatimonadetes* and *Chlorobi* that were not found in the rumen fluid of high‐yielding dairy cows.

**Table 3:** Significant differences in rumen fluid flora between high‐yield dairy cows and Bohai Black at the phylum level

Phylum	Mean (DH)	*SE* (DH)	Mean (H)	*SE* (H)	*p*‐Value	FDR
Bacteroidetes	45.0223	3.7867	54.7922	2.0628	0.0453	0.0629
Proteobacteria	33.2399	5.7075	1.5127	0.1838	0.0008	0.0026
Firmicutes	15.0857	1.1441	34.9734	1.8637	0.0001	0.0011
Spirochaetes	2.5066	0.6484	0.8787	0.0311	0.0300	0.0441
Cyanobacteria	1.4165	0.1463	0.2026	0.0386	0.0003	0.0013
Tenericutes	0.8319	0.1803	1.8821	0.0867	0.0011	0.0029
Verrucomicrobia	0.2051	0.0898	2.4551	0.2315	0.0002	0.0011
Euryarchaeota	0.1030	0.0301	0.7138	0.1168	0.0012	0.0029
Planctomycetes	0.0302	0.0077	0.2654	0.0199	0.0001	0.0011
Synergistetes	0.0266	0.0061	0.0804	0.0104	0.0022	0.0046
Chloroflexi	0.0125	0.0090	0.8927	0.0661	0.0000	0.0000
Actinobacteria	0.0073	0.0019	0.0330	0.0034	0.0004	0.0016
WPS‐2	0.0047	0.0017	0.0219	0.0047	0.0061	0.0109
Lentisphaerae	0.0042	0.0023	0.1175	0.0315	0.0051	0.0099
LD1	0.0003	0.0003	0.0517	0.0189	0.0202	0.0316
Armatimonadetes	0.0000	0.0000	0.0211	0.0033	0.0005	0.0017
Chlorobi	0.0000	0.0000	0.0027	0.0009	0.0093	0.0156

Note

*p < *0.05 denotes significant differences. DH represents rumen fluid of high‐yield dairy cows, and H represents rumen fluid of Chinese Bohai Black cattle.

It can be seen from Table 4 that the rumen fluid in high‐yield dairy cows contained significantly higher levels of* Proteobacteria*,* Spirochaetes,* and *Cyanobacteria* than the rumen fluid of Chinese Luxi Yellow cattle (*p* < 0.05). The relative contents of *Bacteroidetes*,* Firmicutes*,* Tenericutes*,* Verrucomicrobia*,* Euryarchaeota*,* Planctomycetes*,* Green Bacteroides*,* Chloroflexi*,* Actinobacteria*, WPS‐2, *Lentisphaerae,* and LD1 were significantly lower in the rumen fluid of high‐yield dairy cows than in the rumen fluid of Chinese Luxi Yellow cattle (*p* < 0.05). The rumen fluid of Chinese Bohai Black cattle contained low abundance *Armatimonadetes* and *Chlorobi* that were not found in the rumen fluid of high‐yielding dairy cows.

**Table 4:** Significant differences in rumen flora between high‐production dairy cows and Luxi cattle at the level of phylum

Phylum	Mean (DH)	*SE* (DH)	Mean (Y)	*SE* (Y)	*p*‐Value	FDR
Bacteroidetes	45.0223	3.7867	54.4212	1.2628	0.0419	0.0616
Proteobacteria	33.2399	5.7075	1.0414	0.1749	0.0004	0.0011
Firmicutes	15.0857	1.1441	36.5033	0.7948	0.0000	0.0000
Spirochaetes	2.5066	0.6484	0.6748	0.0857	0.0220	0.0344
Cyanobacteria	1.4165	0.1463	0.1900	0.0448	0.0001	0.0008
Tenericutes	0.8319	0.1803	2.1677	0.2656	0.0028	0.0050
Verrucomicrobia	0.2051	0.0898	1.6561	0.1756	0.0002	0.0009
Euryarchaeota	0.1030	0.0301	0.5191	0.0828	0.0018	0.0034
Planctomycetes	0.0302	0.0077	0.2794	0.0348	0.0003	0.0010
Chloroflexi	0.0125	0.0090	0.8676	0.1580	0.0006	0.0013
Actinobacteria	0.0073	0.0019	0.0492	0.0040	0.0001	0.0007
WPS‐2	0.0047	0.0017	0.0239	0.0059	0.0115	0.0192
Lentisphaerae	0.0042	0.0023	0.1007	0.0127	0.0002	0.0009
LD1	0.0003	0.0003	0.0349	0.0051	0.0003	0.0010
Armatimonadetes	0.0000	0.0000	0.0194	0.0021	0.0000	0.0005
Chlorobi	0.0000	0.0000	0.0044	0.0006	0.0003	0.0009

Note

*p < *0.05 denotes significant differences. DH represents rumen fluid of high‐yield dairy cows, and Y represents rumen fluid of Chinese Luxi Yellow cattle.

##### Genus‐level identification

**Figure 8:**
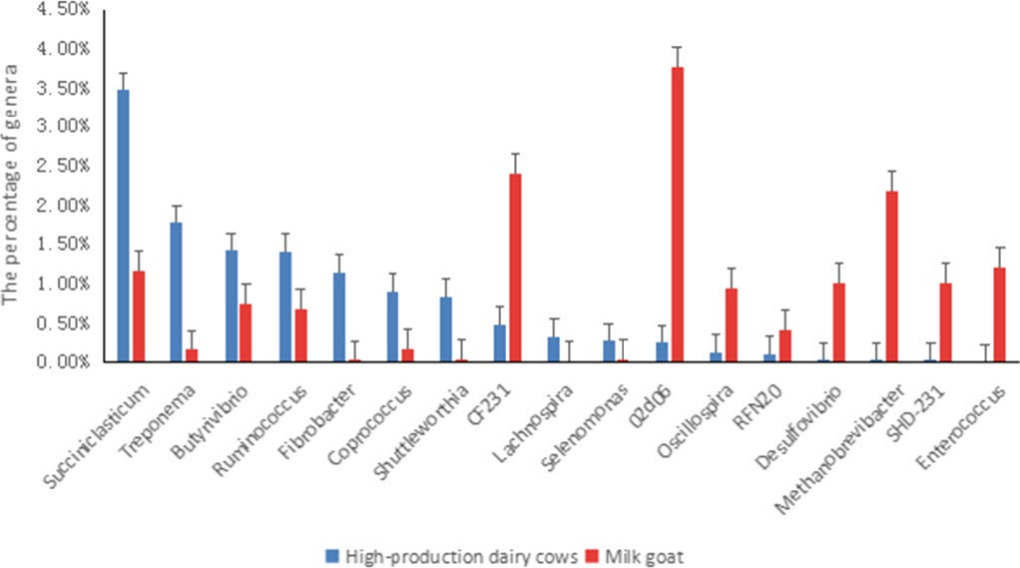
Significant difference in rumen flora between high‐production dairy cows and milk goat at the genus level

The proportion of unidentified genera accounted for 46.11%, 30.93%, 43.64%, 46.90%, and 53.19% of bacteria in the rumen fluid samples of high‐yield dairy cows, low‐yield dairy cows, dairy goats, Chinese Bohai Black cattle, and Chinese Luxi Yellow cattle, respectively. The genera present in proportions >0.1% were determined for all the rumen samples (Figure 8).

Compared with the rumen fluid of low‐yield dairy cows, that of high‐yield dairy cows contained, a higher proportions of *Butyrivibrio, Lachnospira, and Dialister* (*p* < 0.05), and a lower proportions *of Prevotella, Succiniclasticum, Ruminococcus, Coprococcus,* YRC22, CF231, 02d06*, Anaeroplasma Selenomonas,* and *Ruminobacter* (*p* < 0.05; Figure 8).

As shown in Figure 8, compared with rumen fluid of dairy goats rumen fluid, that of high‐yielding dairy cows showed a higher proportions of *Succiniclasticum, Treponema, Butyrivibrio, Ruminococcus, Fibrobacter, Coprococcus, Shuttleworthia, Helicobacter, Lachnospira,* and *Selenomonas* (*p* < 0.05), and a lower proportions of CF231, 03d06,* Oscillobira,* RFN20*, Desulfovibrio, Methanobrevibacter,* SHD‐231, and *Enterococcus* (*p* < 0.05).

**Figure 9:**
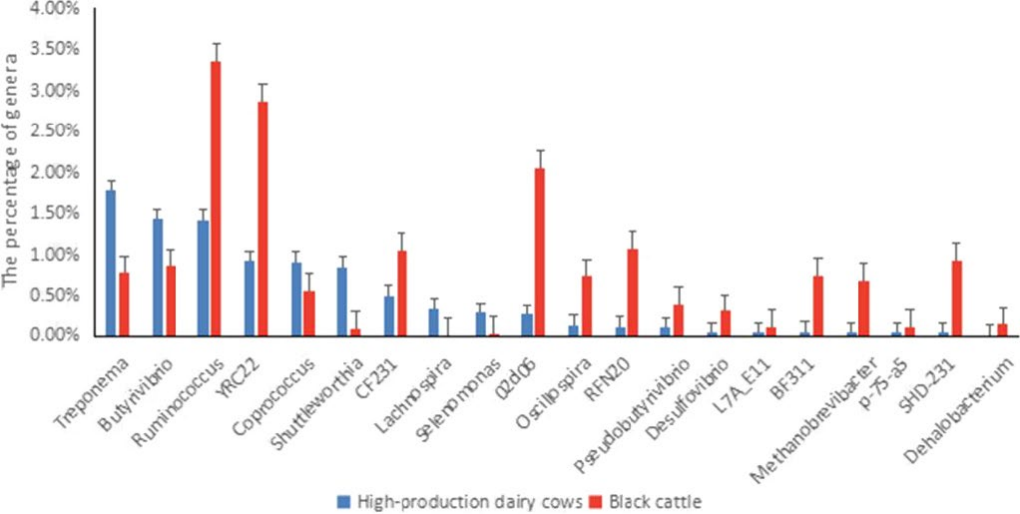
Significant differences in rumen fluid flora between high‐yield dairy cows and Chinese Bohai Black cattle at the genus level

As shown in Figure 9, compared with the rumen fluid of Chinese Bohai Black cattle, the rumen fluid of the high‐yield dairy cows displayed a significantly higher proportions of *Treponema, Butyrivibrio, Coprococcus, Shuttleworthia, Lachnospira,* and *Selenomonas* (*p* < 0.05), and a significantly lower proportions of *Ruminococcus,* YRC22, CF231, 02d06*, Oscillospira,* RFN20,* Pseudobutyrivibrio, Desulfovibrio,* L7A_E11, BF311,* Brevibacterium, Methanobrevibacter,* p‐75‐a5, SHD‐231, and *Dehalobacterium* (*p* < 0.05).

**Figure 10:**
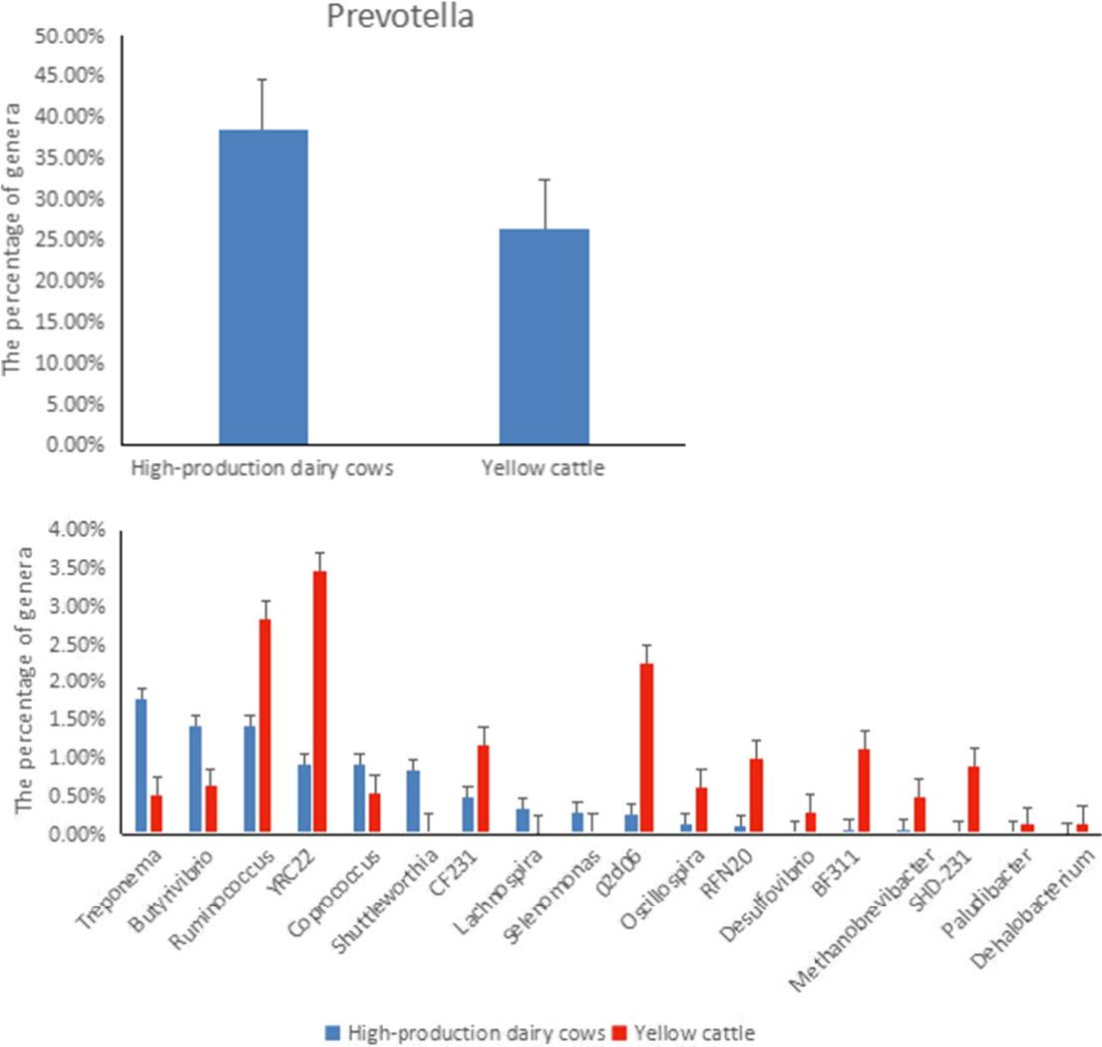
Significant differences in rumen flora between high‐production dairy cows and Luxi Yellow cattle at the genus level

As shown in Figure 10, compared with the rumen fluid of Luxi cattle, that of high‐yield dairy cattle contained a higher proportions of *Prevotella, Treponema, Butyrivibrio, Sclerotia, Shuttleworthia, Lachnospira,* and *Selenomonas* (*p* < 0.05), and a lower proportions of *Ruminococcus,* YRC22, CF231, 02d06*, Phytophthora, Oscillospira,* RFN20*, Desulfovibrio,* BF311*, Methanobrevibacter,* SHD‐231*, Paludibacter,* and *Dehalobacterium* (*p* < 0.05).

## 4 DISCUSSION

The high milk‐yield Holstein dairy cows were significantly enriched for the genera *Butyrivibrio*,* Lachnospir*a, and *Dialister* when compared with the low milk‐yield Holstein dairy cows. The genera *Butyrivibrio* and *Lachnospira* both belong to the Family* Lachnospiraceae*. In the rumen, some special strains of *Butyrivibrio fibrisolvens* completely and quickly degrade cellulose. *Lachnospira* sp. are mostly involved with pectin degradation (Cotta & Forster, 2006). Lima et al. (2015) revealed a positive correlation between *Butyrivibrio* abundance and milk yield. Jami, White, and Mizrahi (2014) showed a positive correlation between *Dialiste*r and milk yield. *Ruminococcus*,* Coprococcus,* and *Succiniclasticum* were suggested to have a negative impact on milk production (Jami et al., 2014), which was consistent with our results.

Compared with the rumen fluid of Chinese Luxi Yellow cattle, Chinese Laoshan dairy goats, and Chinese Bohai Black cattle, the rumen fluid of the high milk‐yield Holstein dairy cows displayed a significantly lower proportions of CF231, *Oscillospira*, RFN20, *Desulfovibrio*,* Methanobrevibacter,* and SHD‐231 (*p* < 0.05). Compared with the low milk‐yield dairy cows, the high milk‐yield Holstein dairy cows contained a lower proportions of *Prevotella*,* Succiniclasticum*,* Ruminococcus*,* Coprococcus*, YRC22, CF231, 02d06, *Anaeroplasma Selenomonas,* and *Ruminobacte*r (*p* < 0.05).

We hypothesize that we can transplant *Oscillospira*,* Desulfovibrio,* and *Methanobrevibacter* to the high milk‐yield Holstein dairy cows to improve fiber digestion. Because these bacterial genera exist in the rumen fluid of Chinese Luxi Yellow cattle, Chinese Laoshan dairy goats, and Chinese Bohai Black cattle but not in the rumen fluid of low milk‐yield dairy cows, we believe that these genera can promote the use of roughage in the rumen without reducing milk production of the high milk‐yield Holstein dairy cows. *Oscillospira* is an anaerobic bacterium that is difficult to culture and is commonly found in the gut of herbivores (Mackie et al., 2003). The genus *Oscillospira* contains genes encoding cellulase, hemicellulase, and oligosaccharide‐degrading enzymes, which are closely related to the degradation of plant polysaccharides. It can promote the degradation of plant cellulose by animals and enable animals to more fully utilize refractory plant polysaccharides to obtain energy (2017). The preponderant gut bacteria in larval Protaetia brevitarsis (Coleoptera:Scarabaedia) fed on corn stalk is *Desulfovibrio* (2017). In some herbivores, such as Venezuelan sheep (2008), African wild impala (2015), North American Holstein (2011), and Chinese Dechang buffalo (2014), *Methanobrevibacter* play an important role. *Methanobrevibacter* produce CH_4_ using hydrogen and formic acid, which reduces the concentration of hydrogen in the digestive tract and improves fermentation efficiency; this study found that *Methanobrevibacter* serves as an "energy transfer agent" in the microflora of the digestive tract (2006). Very few studies refer to the other significant bacteria such as RNF20 and SHD‐231, and their functions in rumen physiology remain unknown.

## 5 CONCLUSION

There were significant differences in ruminal flora between the high‐yield dairy cows and Chinese Laoshan dairy goats, Chinese Bohai Black cattle, and Chinese Luxi Yellow cattle, and the differences were mainly reflected in the relative contents of certain taxa.

## CONFLICT OF INTEREST

The authors declare no conflict of interest.

## AUTHORS CONTRIBUTION

Zhang Tian and Mu Yingyu: Collection and assembly of data, data analysis and interpretation, and writing the article. Zhang Deqing: Collection and assembly of data, and data analysis and interpretation. Lin Xueyan and Wang Zhonghua: Research concept and design, corresponding authors. Hou Qiuling, Wang Yun, and Hu Zhiyong: Data analysis and interpretation.

## ETHICS STATEMENT

All management and experimental procedures were conducted according to the Laboratory Animals‐Guideline of welfare and ethics of China.

## Data Availability

Our data have been uploaded to National Center for Biotechnology Information (NCBI). BioProject: PRJNA489133.
